# Clinical, hormonal and pathomorphological markers of somatotroph pituitary neuroendocrine tumors predicting the treatment outcome in acromegaly

**DOI:** 10.3389/fendo.2022.957301

**Published:** 2022-09-16

**Authors:** Agnieszka Tomasik, Maria Stelmachowska-Banaś, Maria Maksymowicz, Izabella Czajka-Oraniec, Dorota Raczkiewicz, Grzegorz Zieliński, Jacek Kunicki, Wojciech Zgliczyński

**Affiliations:** ^1^Department of Endocrinology, Centre of Postgraduate Medical Education, Warsaw, Poland; ^2^Department of Cancer Pathomorphology, Maria Sklodowska-Curie National Research Institute of Oncology, Warsaw, Poland; ^3^Department of Medical Statistics, School of Public Health, Centre of Postgraduate Medical Education, Warsaw, Poland; ^4^Department of Neurosurgery, Military Institute of Medicine, Warsaw, Poland; ^5^Department of Neurosurgery, Maria Sklodowska-Curie National Research Institute of Oncology, Warsaw, Poland

**Keywords:** pathological results, somatotroph tumors, treatment outcome, acromegaly, surgical remission, response to somatostatin receptor ligands

## Abstract

**Background:**

Transsphenoidal adenomectomy (TSS) of somatotroph pituitary neuroendocrine tumor (PitNET) is the first-line treatment of acromegaly. Pharmacological treatment is recommended if surgery is contraindicated or did not lead to disease remission. The choice of treatment best fitting each patient should be based on thorough investigation of patients’ characteristics. The current analysis attempts to create a tool for personalized treatment planning.

**Aim:**

This study aimed to assess whether clinical, biochemical, imaging and pathological characteristics can predict surgical remission and response to first-generation somatostatin receptor ligands (SRLs) and pasireotide-LAR in acromegaly.

**Patients and methods:**

A retrospective study of 153 acromegaly patients, treated in the Department of Endocrinology in Bielanski Hospital in Warsaw, Poland was performed. Data on demographics, hormonal and imaging results, pathological evaluation, and treatment outcome was extracted from the Polish Acromegaly Registry collecting information from 11 endocrinology centers in Poland and analyzed.

**Results:**

Patients with surgical remission had lower GH and IGF-1 concentrations at diagnosis (median GH 5.5 µg/L [IQR: 3.1-16.0] *vs*. 19.9 µg/L [IQR: 9.8-42.4], p=<0.001 and mean IGF-1 3.1xULN ± SD=1.2 vs. 3.7xULN ± SD=1.2, p=0.007, respectively) and smaller tumors (median 12.5mm [IQR: 9-19] vs. 23mm [IQR: 18-30], p<0.001). These tumors were more often densely granulated (DG) (73.2% vs. 40.0%, p=0.001) with positive staining for alpha-subunit (α-SU) (58.3% vs. 35.5%, p=0.021) and lower Ki-67 index (p=0.002). Patients responding well to SRLs were more often male (55.6% vs 44.4%, p=0.026), presented lower GH concentration (median GH 17.2 µg/L [IQR: 6.2-29.0] vs. 23.8 µg/L [IQR: 11.2-49.5], p=0.048) and had more often DG tumors (63.0% vs. 14.3%, p<0.001). No significant differences between good and poor-response to pasireotide-LAR groups were found. In multivariate logistic regression analysis fasting GH concentration <8.63 µg/L, maximal tumor diameter <15.5mm, normoprolactinemia and DG tumor turned out to be independent predictors of surgical remission (OR=0.92, p=0.026; OR=0.87, p=0.069, OR=3.86, p=0.096 and OR=3.05, p=0.181, respectively). Fasting GH concentration <36.6 µg/L and DG tumor turned out to be independent predictors of good response to first-generation SRLs (OR=0.96, p=0.06 and OR=10.68, p=0.002, respectively).

**Conclusions:**

Younger age at diagnosis, male sex, lower GH, IGF-1 and PRL concentrations, smaller tumor size at diagnosis as well as positive α-SU staining, lower Ki-67 index and DG tumors predicted better treatment outcome in acromegaly patients.

## Introduction

Acromegaly is an insidious rare disease caused by a somatotroph pituitary neuroendocrine tumor (PitNET). Uncontrolled acromegaly is associated with a twofold risk of mortality comparing to the general population, due to mainly neoplastic disease, but also cardiovascular, respiratory, and metabolic complications (1, 2). Over the past decade, disease outcome has improved as a result of enhanced therapeutic strategies, leading to reversal of the increased mortality risk traditionally associated with acromegaly (2, 3). The risk of complications and comorbidities in acromegaly is lower in patients who are biochemically controlled (4, 5). Recent studies have shown that the mortality rate in controlled acromegaly is similar to the general population (1).

Treatment of acromegaly is aimed at resecting the disease-causing lesion and reducing GH and IGF-1 levels to normal values which results in normal-life expectancy and improvement in comorbidities. Transsphenoidal adenomectomy (TSS) is considered a first-line treatment giving a chance for immediate cure in some patients and amelioration of disease manifestations in most patients (6). However, the surgical cure rates vary between 32 and 85% depending on various factors such as tumor size, cavernous sinus invasion, surgeon experience and the remission criteria used (7–10). Some other factors such as preoperative growth hormone (GH) and insulin-like growth factor-1 (IGF-1) concentrations, sex, diagnosis age and intensity of T2-weighted signal in magnetic resonance imaging (MRI) scan are considered to predict surgical treatment outcome (11–13). Pathological assessment of postoperative tissue including immunohistochemistry (IHC) staining for anterior pituitary hormones, Ki-67 index or granulation pattern, may provide clinicians with valuable information on tumor’s characteristics, which might help to predict the disease course and treatment outcome. In routinely performed IHC, alpha-subunit (α-SU) staining is assessed. So far, there have been trials focused on α-SU serum concentration in acromegaly (14, 15). However, data on the meaning of α-SU staining in post-operative somatotroph tumor specimen is lacking. In patients with non-complete surgery medical treatment with first-generation somatostatin receptor ligands (SRLs) is recommended, but the efficacy is moderate (IGF-1 normalization is achieved in about 30% of patients) (16). Some clinical factors e.g. age, sex, IGF-1 concentration, body weight, as well as T2-weighted signal in MRI scan might be predictive of remission on first-generation SRLs (17–19). Moreover, it has been previously reported that histologic characteristics such as granulation pattern are predictive of tumor response to medical therapies (20, 21). Pasireotide long-acting release (pasireotide-LAR) is one of the second-line drugs, recommended for patients with resistance to first-generation SRLs. So far it has been shown that T2-weighted signal in MRI, SSTR expression, AIP expression and Ki-67 index may be predictive of pasireotide-LAR response (22–27). However, there are not many studies to date which have identified clinical and pathomorphological factors predicting the effectiveness of pasireotide treatment.

The need for personalized treatment of acromegaly led to searching for predictors of good surgical and medical treatment outcome. The aim of this study was to identify clinical, hormonal, imaging and pathomorphological variables associated with surgical remission and first- and second- generation SRLs effectiveness in acromegaly and to develop a predictive model of treatment outcome.

## Material and methods

### Patients’ selection

The study cohort was selected from the group of 178 patients with acromegaly treated in the Department of Endocrinology of the Center of Postgraduate Medical Education in Bielanski Hospital in Warsaw, Poland and included in the Polish Acromegaly Registry.

The Polish Acromegaly Registry is a nationwide database established in 2017 with the aim to gather specific data about patients with acromegaly in Poland. The collected data covered epidemiology, diagnosis of acromegaly, hormonal and imaging results, treatment procedures and their effectiveness, co-morbidities and mortality. Until November 2021 eleven academic centers have participated in the Polish Acromegaly Registry and 390 patients have been included. The project received the approval of the Bioethics Committee of the Center of Postgraduate Medical Education in Warsaw, Poland.

**Figure 1:**
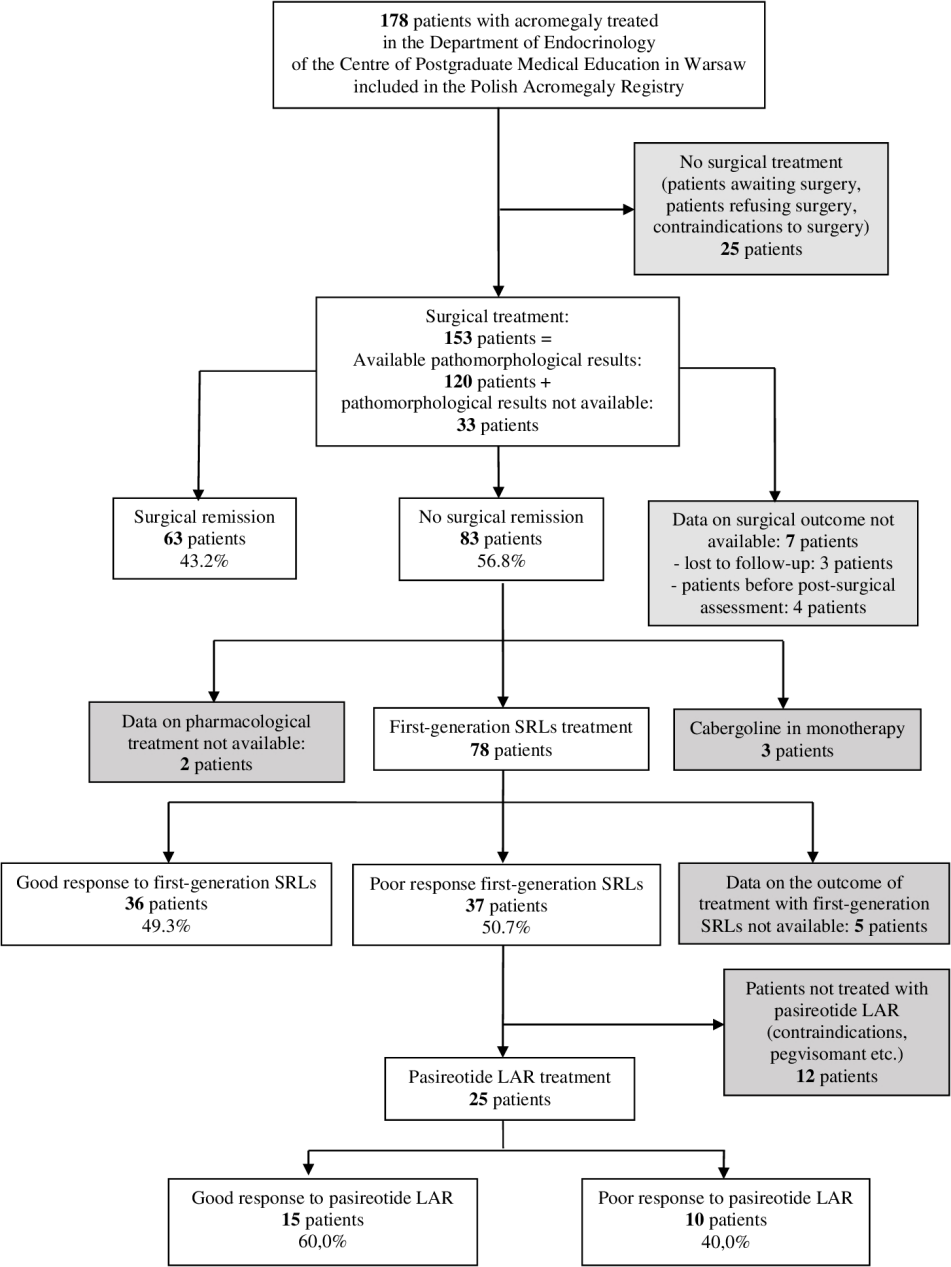
Flowchart of patients cohorts.

One hundred fifty-three patients with acromegaly who were surgically treated between February 2000 and September 2021 were selected from the whole group of 178 patients treated in the Department of Endocrinology of Bielanski Hospital in Warsaw and included in the current retrospective study. Unoperated patients (n=25) were excluded from the study cohort due to possible differences in SRLs’ effectiveness compared with operated patients (28–30). Demographic data, patients’ characteristics at diagnosis including hormonal results and imaging features, treatment outcome as well as pathological results were extracted from the Polish Acromegaly Registry. The MRI examination of the sella turcica region with contrast enhancement in T1- and T2-weighted signal was performed at diagnosis. The MRI scans were evaluated by radiologists experienced in neuroradiology. T2-weighted signal was compared to temporal lobe grey matter and assessed visually. However, due to unavailable data in a large proportion of patients, T2-weighted signal MRI scans were not assessed in the current study. The imaging characteristics covered maximal tumor size expressed as maximal tumor diameter and features of tumor’s invasiveness i.e. extrasellar expansion, compression of the optic chiasm and degree of cavernous sinus invasion (no invasion, unilateral or bilateral invasion). The study cohort selection together with treatment procedures and outcomes are presented in **Figure 1**.

### Treatment procedures

All patients included in the study operated after 2006 (133 patients) were preoperatively treated with first-generation SRLs according to local guidelines (31, 32). The other 20, operated in 2006 or before were pharmacologically naïve before surgery. TSS was performed in 146 patients (96.7%) and transcranial approach was chosen in 7 patients. All patients were operated on by one of the two neurosurgeons experienced in pituitary surgeries, either from the Department of Neurosurgery, Military Institute of Medicine in Warsaw, Poland or from the Department of Neurosurgery, Maria Sklodowska-Curie National Research Institute of Oncology in Warsaw, Poland. Surgical remission was defined as IGF-1 concentration below the upper limit of normal (ULN) for sex and age-matched groups and suppression of GH on oral glucose tolerance test (OGTT) below 1 µg/L assessed 3 - 6 months after pituitary surgery (33). The majority of patients were operated once (139 patients – 90.8%). For reoperated patients pathomorphological results and clinical data after the last surgery were analyzed. The mean time of follow-up after the last surgery was 106 ± 85 months. Response to the medical therapy was assessed after at least a 6-month treatment period. Good response to first-generation SRLs was defined as fasting GH ≤2.5 µg/L and IGF-1 ≤1.2 xULN and poor response to SRLs was defined as GH >2.5 µg/L and IGF-1 >1.2 xULN on treatment with maximum tolerated dose of first-generation SRLs. Eighteen patients received combined treatment with dopamine agonists and first-generation SRLs. The mean time of SRLs treatment was 84 ± 69 months.

Most of the patients without biochemical control of acromegaly on first-generation SRLs were switched to pasireotide-LAR or pegvisomant, when they became reimbursed in Poland, in 2018 and 2020, respectively.

Good response to pasireotide-LAR was defined as fasting GH ≤2.5 µg/L and IGF-1 ≤1.2 xULN assessed after at least a 6-month period of treatment with maximum tolerated dose of pasireotide-LAR, whereas poor response to pasireotide-LAR was defined as fasting GH >2.5 µg/L or GF-1 >1.2 xULN. The mean time of pasireotide-LAR treatment was 29 ± 11 months.

Treatment with pegvisomant was not included in this analysis.

Blood samples from fasting patients taken for GH and IGF-1 in 2011 and before were analyzed using isotope methods: immunoradiometric and radioimmunoassay, respectively. The intra-assay coefficient of variation (CV) is 0.7% for a GH concentration of 4.9 ng/mL and inter-assay CV is 8.1% for a GH concentration of 3.8 ng/mL. The intra-assay CV is 9.6% for an IGF-1 concentration of 160.8 ng/mL and inter-assay CV is 10.4% for an IGF-1 concentration of 172.0 ng/mL. Blood samples taken for GH and IGF-1 after 2011 were analyzed with chemiluminescence immunoassay using the LIAISON^®^ XL analyzer (DiaSorin, Italy). The GH assay has a sensitivity of 0.05 ng/mL, an intra-assay coefficient of variation (CV) of 1.93% for a GH concentration of 1.18 ng/mL and inter-assay CV of. 3.77% for a GH concentration of 1.11 ng/mL. The intra-assay CV is 4.59% for an IGF-1 concentration of 189.3 ng/mL and inter-assay CV is 4.3% for an IGF-1 concentration of 202.6 ng/mL.

### Pathological evaluation

The pathomorphological diagnosis of PitNETs was performed by one experienced pathologist in the Department of Cancer Pathomorphology, Maria Sklodowska-Curie National Research Institute of Oncology, Warsaw, Poland.

Fragments of tumor tissue taken intraoperatively were routinely fixed in 10% buffered formalin, embedded in paraffin and stained with hematoxylin and eosin.

IHC staining was performed on paraffin sections using the EnVision™FlexVisualization System with DAB (3,3’-diaminobenzidine) as chromogen (K8000, Dako/Agilent) using primary antibodies against anterior pituitary hormones: GH, PRL, ACTH, β-TSH, β-FSH, β-LH and α-SU. IHC studies were performed over a period of approximately 20 years, and during this time, antibodies from a variety of companies were used, including: Thermo Scientific, Dako, NeoMarkers, Novocastraor BIO-RAD. The antibodies for Ki-67 were obtained from Dako (MIB-1clone, ready to use antibody) and anti-cytokeratin antibody from Ventana (Cam 5.2, ready to use antibody). The exact description of the staining methods was previously published (34). In selected cases at the request of clinicians, especially in young patients, in patients with aggressive tumors, in patients resistant to first-line treatment or at suspicion of hyperplasia, immunostaining was extended to the expression of SSTR2A, SSTR5, Cam 5.2, p53, MGMT, collagen IV and others.

All staining procedures were carried out at Dako Autostainer Link48.

Transcription factors were not evaluated because most patients were operated before 2017, when such evaluation was not required in routine pathomorphological evaluation.

All the tissue samples were stained in the perioperative period and newly evaluated for the purposes of the current study.

### Electron microscopy

**Figure 2:**
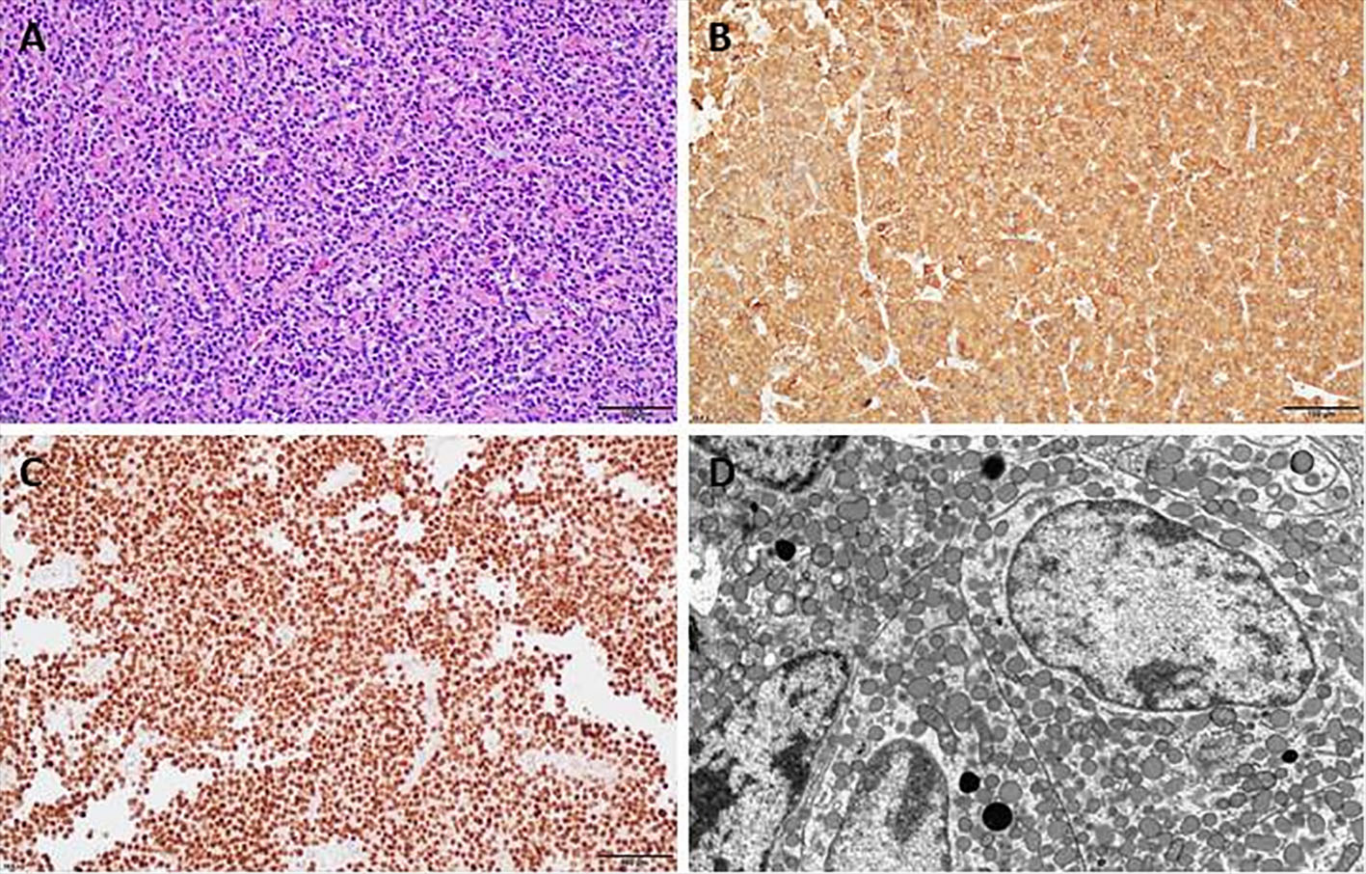
Densely granulated somatotroph pituitary adenoma (PitNET). **(A)** Histology of densely granulated somatotroph PitNET (HE), **(B)** Immunopositive staining for growth hormone (GH), **(C)** Immunopositive staining for PIT1, **(D)** Ultrastructural features of densely granulated somatotroph PitNET.

**Figure 3:**
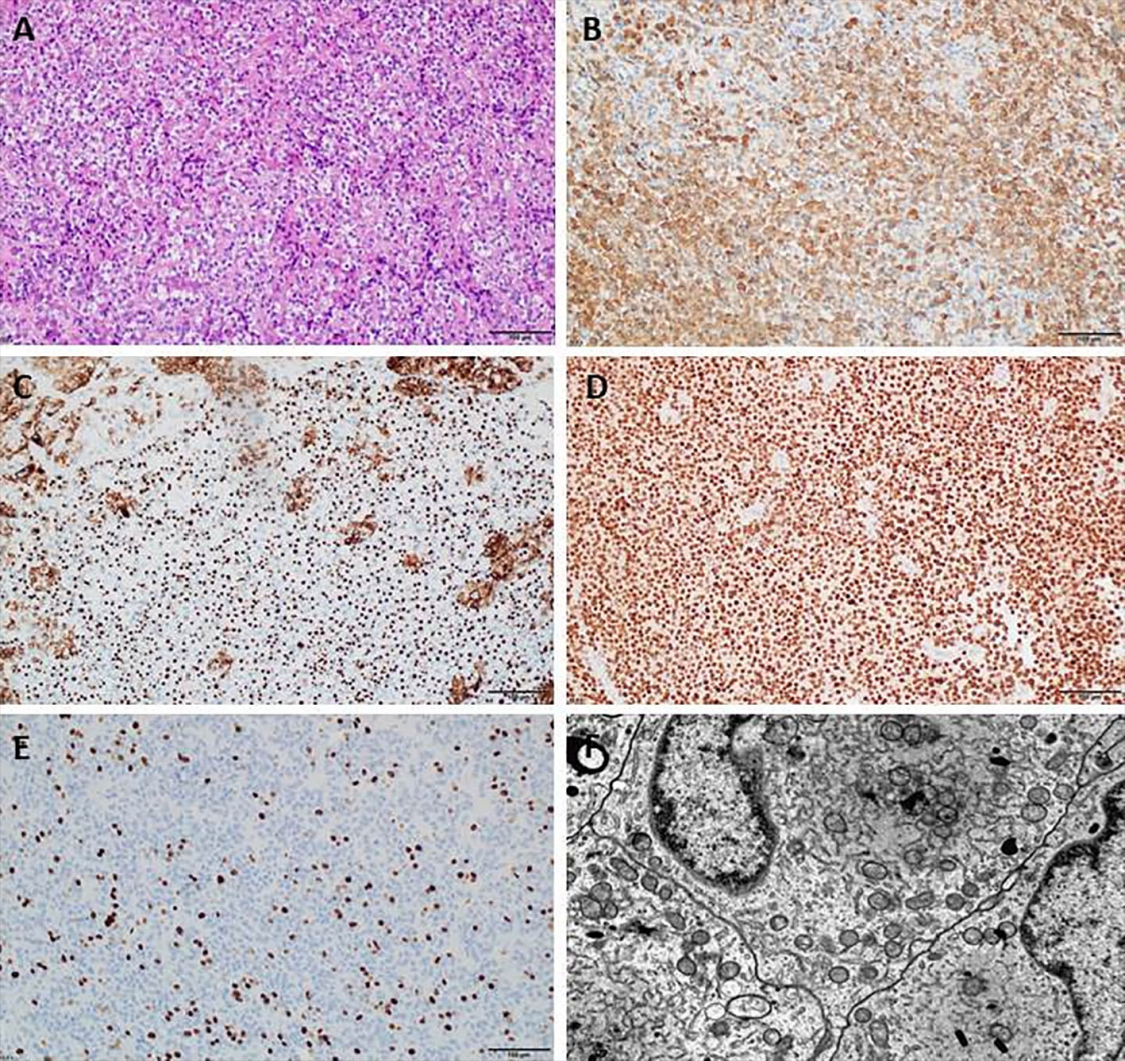
Sparsely granulated somatotroph pituitary adenoma (PitNET). **(A)** Histology of sparsely granulated somatotroph PitNET (HE), **(B)** Immunopositive staining for growth hormone (GH), **(C)** Immunopositive staining for Cam5.2, **(D)** Immunopositive staining for PIT1, **(E)** Immunopositive staining for Ki-67, **(F)** Ultrastructural features of sparsely granulated somatotroph PitNET.

For 108 patients EM was performed. In other cases, no material for EM was obtained. Small pieces of pituitary tumor tissues were fixed in 2.5% glutaraldehyde and postfixed in 1% osmium tetroxide, dehydrated with ethanol and propylene oxide and subsequently embedded in epoxy resin (Epon 812). Ultrathin sections were counterstained with uranyl acetate and lead citrate and examined with a Philips CM120 BioTWIN transmission EM. Somatotroph tumors were classified on the basis of commonly accepted histological and ultrastructural features, such as densely granulated (DG) or sparsely granulated (SG) (35). The examples of DG and SG tumors are visualized in **Figures 2**, **3**.

### Statistical analysis

The data were analyzed using STATISTICA 13 and SPSS software.

Absolute numbers (n) and percentages (%) of the occurrence of categories were estimated for categorical variables. Normal distribution of continuous variables was verified with Shapiro-Wilk’s test and based on visual assessment of histograms. Mean (M) and standard deviation (SD) were estimated for normally distributed continuous variables. Median (Me) with interquartile range (IQR) for non-normally distributed continuous variables.

To compare clinical, imaging or pathological variables between a group of patients with surgical remission and without surgical remission, between a group of patients responding well and poor to first-generation SRLs, between a group of patients responding well and poor to pasireotide-LAR, the following statistical tests were used:

* Student’s t-test to compare normally distributed continuous variables;
* Mann-Whithey’s U-test to compare non-normally distributed continuous variables;
* Pearson’s chi-square test to compare categorical variables if all the expected counts were at least equal 5;
* Fisher’s exact test to compare categorical variables if any expected count were smaller than 5.

A significance level was assumed to be 0.05 in all the statistical tests.

If a significant association between surgical remission and clinical, imaging or pathological variables was found, an odds ratio (OR) of surgical remission was estimated using binary logistic regression analysis. If many significant predictors of surgical remission were found, multivariate binary logistic regression analysis was performed, in order to select only independent predictors of surgical remission using backward selection method. A significance level was assumed to be 0.20 in all the logistic regression models. The same was performed to response to medical treatment. Sensitivity, specificity and percentage of correctly classified patients were calculated as well as a receiver operating curve (ROC) was drawn for logistic regression model of surgical remission and good response to first-generation SRLs. Cut-off point for continuous predictors was estimated based on Kolmogorow-Smirnow statistics, i.e. maximum difference between the sensitivity and 1-specificity in ROC analysis.

Missing data were omitted in statistical analysis.

## Results

### Clinical characteristics and hormonal results

Clinical characteristics, hormonal results and their impact on surgical remission and medical treatment are presented in **Table 1**.

**Table 1:** Clinical presentation of the study cohort.

Variable, parameter	Unit or category	Total		Surgical remission					Response to first-generation SRLs				
				Yes		No		p	Good		Poor		p
		N	Results	N	Results	N	Results		N	Results	N	Results	
Age, M ± SD	years	153	53.5 ± 12.5	63	56.8 ± 11.0	83	50.6 ± 13.1	**0.003**	36	51.5 ± 12.2	37	49.8 ± 13.9	0.577
Sex, n (%)	Males	153	65 (42.5)	63	28 (44.4)	83	34 (41.0)	0.673	36	20 (55.6)	37	11 (29.7)	**0.026**
	Females		88 (57.5)		35 (55.6)		49 (59.0)			16 (44.4)		26 (70.3)	
Age at diagnosis, M ± SD	years	153	43.8 ± 12.7	63	48.9 ± 11.0	83	39.0 ± 12.1	**<0.001**	36	39.8 ± 11.4	37	38.6 ± 12.7	0.677
Diagnostic delay, Median [IQR]	years	145	8 [4-18]	58	9 [4-20]	80	8 [3-18]	0.658	35	8 [4-20]	35	5 [3-18]	0.417
Fasting GH at diagnosis, Median [IQR]	µg/L	132	11.9 [4.3-26.0]	55	5.5[3.1-16.0]	70	19.9 [9.8-42.4]	**<0.001**	29	17.2 [6.2-29.0]	32	23.8 [11.2-49.5]	**0.048**
IGF-1 at diagnosis, M ± SD	ng/mL	131	874.7 ± 321.3	57	751.2 ± 285.7	68	994.0 ± 314.0	**<0.001**	28	1006.8 ± 362.3	33	992.2 ± 298.6	0.863
IGF-1 at diagnosis, M ± SD	x ULN	130	3.4 ± 1.2	57	3.1 ± 1.2	67	3.7 ± 1.2	**0.007**	28	3.8 ± 1.2	32	3.7 ± 1.2	0.771
PRL at diagnosis, Median [IQR]	ng/mL	105	13.7 [8.8-27.7]	44	11.1[9.2-15.0]	55	19.7 [7.8-38.8]	**0.026**	20	20.1 [8.8-48.9]	28	21.3 [7.5-32.3]	0.714
Hyperprolactinaemia at diagnosis, n (%)	yes	105	33 (31.4)	44	7 (15.9)	55	25 (45.5)	**0.002**	20	9 (45.0)	28	13 (46.4)	0.922
Visual field defects, n (%)	None	153	128 (83.7)	63	57 (90.5)	83	64 (77.1)	0.091	36	30 (83.3)	37	24 (64.9)	**0.028**
	Small		14 (9.2)		4 (6.4)		10 (12.1)			1 (2.8)		9 (24.3)	
	Quadrantanopia		11 (7.2)		2 (3.2)		9 (10.8)			5 (13.9)		4 (10.8)	

N – number of patients for whom data were available. The results are presented as n (%) for categorical variables, M ± SD for normally distributed continuous variables, Median [IQR] for non-normally distributed continuous variables. M, mean; SD, standard deviation; IQR, interquartile range. The bold values are statistically significant.

Among 153 patients, 88 patients were females (57.5%). The mean BMI was 29.3 kg/m^2^ ± 4.9. The mean age at diagnosis of acromegaly was 43.8 years ± 12.7 and the median diagnostic delay was 8 years (IQR: 4-18). At diagnosis, the median fasting GH concentration was 11.9 µg/L (IQR: 4.3 - 26.0), the mean IGF-1 concentration was 3.4 x ULN ± 1.2 and approximately 31% of patients had hyperprolactinemia. Thirty-one percent of patients had at least one pituitary axis dysfunction at diagnosis, most often gonadotropins deficiency (22.2%), followed by corticotropin deficiency (8.5%) and thyrotropin deficiency (5.9%). Genetic syndromes causing acromegaly were clinically diagnosed in 12 patients (7.8%): MEN1 syndrome in 6 patients, familial isolated pituitary adenomas (FIPA) in 4 patients and McCune-Albright syndrome in 2 patients. Twenty-five patients (16.3%) presented visual field defects, 11 of them were affected by quadrantanopia (7.2%).

Sixty-three patients achieved surgical remission (43.2%) and 83 patients did not (56.8%). Data on surgical outcome was not available for 7 patients (**Figure 1**). Patients with surgical remission were older at diagnosis of acromegaly (49 years ±11 vs. 39 years ±12, p<0.001], had lower fasting GH [5.5 µg/L (IQR: 3.1-16.0) vs. 19.9 µg/L (IQR: 9.8-42.4), p=0.007], lower IGF-1 (3.1 xULN ± 1.2 vs. 3.7 xULN ± 1.2) and lower PRL concentrations at diagnosis [11.1 ng/mL (IQR: 9.2-15.0) vs. 19.7 ng/mL (IQR: 7.8-38.8), p=0.026]. They presented with hyperprolactinemia and at least one pituitary axis dysfunction at diagnosis less often (15.9% vs. 45.5% and 22.2% vs. 38.6%, p=0.002 and p=0.035, respectively) than patients with active acromegaly after surgery. Patients without surgical remission had more often gonadotropins and thyrotropin deficiency at diagnosis when compared to patients in remission-group (30.1% vs. 12.7% and 9.6% vs. 1.6%, p=0.013 and p=0.044, respectively). They were also more frequently diagnosed with genetic syndromes associated with acromegaly compared to patients with surgical remission (12.1% vs. 1.6%, p=0.024). Sex, diagnostic delay and frequency of visual field defects did not differ in both groups.

Seventy-eight patients out of 83, who did not achieve surgical remission, were treated with first-generation SRLs postoperatively. Thirty-six of them responded to SRLs well and 37 - poorly according to the accepted criteria. The data on the outcome of first-generation SRLs treatment was not available for 5 patients (**Figure 1**). Males and patients with lower fasting GH concentration at diagnosis responded better to first-generation SRLs (55.6% vs. 44.4%, p=0.026 and 17.2 µg/L [IQR: 6.2-29.0] vs. 23.8 µg/L [IQR: 11.2-49.5] p=0.048, respectively). Patients without visual field defects also presented better response to first-generation SRLs (83.3% vs. 64.9%, p=0.028). Moreover, we observed a strong tendency towards statistical significance for normal hypothalamic-pituitary-thyroid-axis function at diagnosis in good-response to SRLs group (p=0.054). Age at diagnosis, diagnostic delay, IGF-1 and PRL concentration did not affect the response to first-generation SRLs.

Twenty-five out of 37 patients, who did not achieve good response to first-generation SRLs, were treated with pasireotide-LAR. Fifteen patients achieved good response to pasireotide-LAR and 10 patients did not according to defined criteria. There were no statistically significant differences among analyzed clinical factors and hormonal results between good-response to pasireotide-LAR and poor-response group.

### Imaging features of tumors

The imaging presentation of tumors in the study cohort is presented in **Table 2**.

**Table 2:** Imaging presentation of the study cohort.

Variable, parameter	Unit or category	Total		Surgical remission					Response to first-generation SRLs				
				Yes		No		p	Good		Poor		p
		N	Results	N	Results	N	Results		N	Results	N	Results	
Tumor imaging in MRI, n (%)	Microadenoma	146	31 (21.2)	58	23 (40.0)	81	6 (7.4)	**<0.001**	35	5 (14.3)	37	1 (2.7)	0.102
	Macroadenoma		115 (78.7)		35 (60.0)		75 (92.6)			30 (85.7)		36 (97.3)	
Maximal tumor diameter, Median [IQR]	mm	124	19 [12-25]	54	12.5 [9-19]	64	23 [18-30]	**<0.001**	26	22 [16-28]	32	25 [20-35]	0.162
Extrasellar expansion, n (%)	yes	129	73 (56.6)	48	14 (29.2)	74	58 (78.4)	**<0.001**	30	18 (60.0)	35	32 [91.4]	**0.003**
Compression of the optic chiasm, n (%)	yes	122	31 (25.4)	48	6 (12.5)	67	25 (37.3)	**0.003**	28	5 (17.9)	32	19 (59.4)	**0.001**
Degree of cavernous sinus invasion, n (%)	None	130	69 (53.1)	53	44 (83.0)	74	22 (29.7)	**<0.001**	30	12 (40.0)	35	8 (22.9)	**0.043**
	Unilateral		47 (36.2)		9 (17.0)		38 (51.4)			16 (53.3)		17 (48.6)	
	Bilateral		14 (10.8)		0 (0.0)		14 (18.9)			2 (6.7)		10 (28.6)	

The bold values are statistically significant.

Most of the patients had macroadenomas on MRI scan (78.7%) with maximal tumor diameter of 19.0 mm on average. Seventy-three tumors (56.6%) showed extrasellar expansion, 31 compressed the optic chiasm (25.4%) leading to visual field defects in 25 patients (16.3%), whereas 61 tumors (46.9%) invaded the cavernous sinuses.

Patients who achieved surgical remission had smaller tumors [12.5mm (IQR: 9-19) vs. 23mm (IQR: 18-30), p<0.001]. Microadenomas in the remission group constituted 40.0% vs. 7.4% in the non-remission group, p<0.001. In patients with surgical remission extrasellar expansion and compression of the optic chiasm were less often (29.2% vs. 78.4%, p<0.001 and 12.5% vs. 37.3%, p=0.003, respectively). Their tumors also presented smaller degree of cavernous sinus invasion (83.0% of patients without cavernous sinus invasion in the remission group vs. 29.7% of patients without cavernous sinus invasion in the non-remission group, p<0.001).

Patients without extrasellar expansion, without compression of the optic chiasm and without cavernous sinuses invasion at diagnosis were more likely to present good response to first-generation SRLs (40.0% vs. 8.6%, p=0.003, 82.1% vs. 40.6%, p=0.001 and 40.0% vs. 22.9%, p=0.043, respectively).

None of the analyzed imaging factors significantly influenced treatment outcome in pasireotide-LAR-treated group. However, it is worth noting, that pasireotide-LAR treated group included 24 patients with macroadenomas and only 1 patient with microadenoma. All tumors, except of one, in pasireotide-LAR-treated group showed extrasellar expansion.

### Pathomorphological evaluation

The pathomorphological evaluation is presented in **Table 3**.

**Table 3:** Pathomorphological presentation of the study cohort.

Variable, parameter	Unit or category	Total		Surgical remission					Response to first-generation SRLs				
				Yes		No		p	Good		Poor		p
		N	Results	N	Results	N	Results		N	Results	N	Results	
Granulation subtype of tumor, n (%)	Densely	108	59 (54.6)	41	30 (73.2)	60	24 (40.0)	**0.001**	25	16 (64.0)	27	4 (14.8)	**<0.001**
	Sparsely		49 (45.4)		11 (26.9)		36 (60.0)			9 (36.0)		23 (85.2)	
Immunohistochemical evaluation of tumor, n (%)	Pure GH(+)	118	63 (53.4)	48	23 (47.9)	63	34 (54.0)	0.847	27	15 (55.6)	29	17 (58.6)	0.927
	GH(+) PRL(+)		46 (39.0)		21 (43.8)		24 (38.1)			10 (37.0)		9 (31.0)	
	Plurihormonal		9 (7.6)		4 (8.3)		5 (7.9)			2 (7.4)		3 (10.3)	
Alpha-subunit, n (%)	(+)	117	55 (47.0)	48	28 (58.3)	62	22 (35.5)	**0.021**	27	11 (40.7)	29	8 (27.6)	0.299
Ki-67 Index, n (%)	Ki-67 < 1	116	89 (76.7)	48	42 (87.5)	61	40 (65.6)	**0.002**	26	18 (69.2)	29	16 (55.2)	0.094
	1 ≤ Ki-67 < 3		16 (13.8)		6 (12.5)		10 (16.4)			6 (23.1)		4 (13.8)	
	Ki-67 ≥ 3		11 (9.5)		0 (0.00)		11 (18.0)			2 (7.7)		9 (31.0)	

The bold values are statistically significant.

IHC evaluation was performed in 118 patients. Sixty-three (53.4%) tumors were positively staining only for GH - pure GH(+) tumors, 46 (39.0%) tumors stained for GH and prolactin (PRL) - mixed GH(+) PRL(+) tumors and 9 (7.6%) tumors were plurihormonal. Patients with GH(+)PRL(+) tumors presented hyperprolactinemia more frequently than patients with GH(+) and plurihormonal tumors, but the difference was not significant (41.7% vs. 26.9%, p=0.148). Staining for α-SU was assessed in 117 patients and 55 patients showed positive immunohistochemical staining for α-SU (47.0%). Ki-67 index was evaluated in 116 patients: 89 patients had Ki-67 index < 1% (76.7%), 16 patients had Ki-67 index between 1 and 3% (13.8%) and 11 patients had Ki-67 index ≥ 3% (9.5%). EM was performed in 108 patients. There was a slight predominance of DG tumors (54.6%) over SG tumors.

Patients with surgical remission more frequently presented DG somatotroph tumors in EM (73.2% vs. 40.0%, p=0.001) and positive staining for α-SU in IHC evaluation (58.3% vs. 35.5%, p=0.021). Ki-67 index in the remission group was significantly lower than in non-remission group (p=0.002).

As far as treatment with first-generation SRLs is concerned, there were more patients with DG tumors in good-response group compared to poor-response group (64.0% vs. 14.8%, p<0.001). A certain trend for Ki-67 index ≥ 3% in poor-response group was also noted, but it did not reach statistical significance (p=0.094).

Among patients treated with pasireotide-LAR no statistically significant differences were found in pathomorphological factors between good- and poor-response group. However, in patients treated with pasireotide-LAR with available pathomorphological result, most tumors were SG (15 tumors out of 20, 75%). Only five tumors out of 20 in this group showed positive staining for α-SU (25%). Over 50% of all patients with Ki-67 index ≥ 3% (6 out of 11) occurred in pasireotide-LAR treated group.

### Predictors of surgical remission and good response to first generation SRLs

A logistic regression analysis was performed to determine clinical, imaging and pathomorphological characteristics increasing the likelihood of surgical remission and good response to first-generation SRLs.

The cut-off value of fasting GH concentration predicting surgical remission was estimated as GH<8.63 µg/L with sensitivity of 79.2% and specificity of 62.5%. The percentage of correctly classified patients was 65.6%. The cut-off value of maximal tumor diameter predicting surgical remission was estimated as 15.5mm with sensitivity of 89.1% and specificity of 63.0%. The percentage of correctly classified patients was 74.6%.

**Figure 4:**
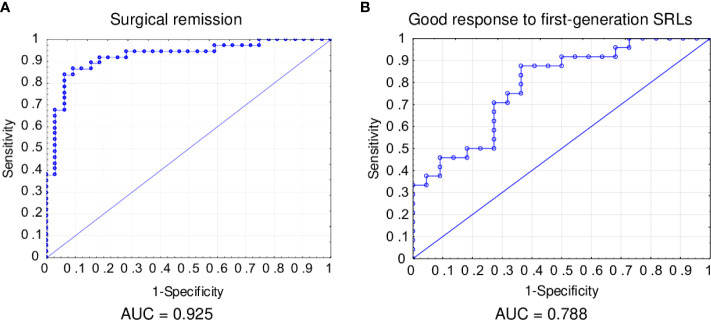
Receiver operating characteristic (ROC) curve for surgical remission **(A)** and good response to first-generation SRLs **(B)**.

In multivariate logistic regression analysis, independent predictors of surgical remission were normoprolactinemia at diagnosis (OR=3.86, p=0.096), DG tumor in EM (OR=3.05, p=0.181), lower fasting GH concentration at diagnosis (OR=0.92, p=0.026) and smaller maximal tumor diameter (OR=0.87, p=0.069). It was estimated with sensitivity of 86.5% and specificity of 84.4% The percentage of correctly classified patients was 85.5% (**Table 4**; **Figure 4A**). The patients with normoprolactinemia at diagnosis had almost 4 times higher the odds of surgical remission, the patients with DG tumor had above 3 times higher the odds of surgical remission. Additionally, the odds of surgical remission were increased by 8.4% if a patient had lower fasting GH concentration at diagnosis by 1µg/L and increased by 13% if a patients had smaller maximal tumor diameter at diagnosis by 1 mm.

**Table 4:** Multivariate logistic regression for surgical remission, n=69.

	OR	p	80% CI for OR	
			Lower limit	Upper limit
Fasting GH at diagnosis [µg/L]	0.916	0.026	0.871	0.963
Maximal tumor diameter [mm]	0.870	0.069	0.789	0,960
Densely vs sparsely granulated subtype of tumor	3.048	0.181	1.048	8.864
Normoprolactinemia vs hyperprolactinemia	3.857	0.096	1.365	10.898

The cut-off value of fasting GH concentration predicting good response to first-generation SRLs was estimated as GH<36.6 µg/L with sensitivity of 45.5% and specificity of 80.0%. The percentage of correctly classified patients was 52.4%.

In multivariate logistic regression analysis, independent predictors of good-response to first-generation SRLs were lower fasting GH concentration at diagnosis (OR=0.96, p=0.06) and DG tumor in EM (OR=10.68, p=0.002). It was estimated with sensitivity of 84.0% and specificity of 63.6%. The percentage of correctly classified patients was 74.5% (**Table 5**; **Figure 4B**). The odds of good-response to first-generation SRLs were increased by 4% if a patient had lower fasting GH concentration at diagnosis by 1µg/L. Patients with DG tumors in EM had above 10 times higher the odds of good-response to first-generation SRLs.

**Table 5:** Multivariate logistic regression for good response to first-generation SRLs, N=47.

	OR	p	80% CI for OR	
			Lower limit	Upper limit
Fasting GH at diagnosis [µg/L]	0.961	0.063	0.935	0.988
Densely vs sparsely granulated subtype of tumor	10.684	0.002	3.968	28.767

No multivariate logistic regression model of good response to pasireotide-LAR was estimated, because no significant differences in analyzed clinical, imaging and pathomorphological characteristics between patients with good and poor response to pasireotide-LAR were found.

## Discussion

In the present study we have shown that older patients, with lower GH, IGF-1 and PRL concentrations, with smaller, less-invasive tumors had a greater chance of surgical remission. Patients of male sex, with lower fasting GH concentration, with tumors without extrasellar expansion, which do not compress the optic chiasm and do not invade cavernous sinuses responded better to first generation SRLs. Moreover, patients with DG somatotroph tumors were more likely to achieve surgical remission and to respond well to first-generation SRLs. Additionally, positive staining for α-SU and lower Ki-67 index increased the likelihood of surgical remission in acromegaly. The best predictors for surgical remission in the current study were fasting GH<8.63 µg/L at diagnosis, maximal tumor diameter below 15.5mm, normoprolactinemia and diagnosis of DG tumor. The best predictors of good response to first-generation SRLs were fasting GH<36.6 µg/L at diagnosis and DG tumor. Our results are of great importance in predicting treatment outcome and personalized treatment planning in acromegaly patients.

It has already been published, that some clinical and imaging characteristics affect treatment effectiveness (5). Some studies have shown that higher preoperative GH concentration, larger pituitary tumor and tumor invasiveness defined as cavernous sinus invasion or high Knosp grade are negative predictors of remission after TSS in acromegaly (36–38). Other significant factors may be age at diagnosis, sex, preoperative IGF-1 concentration or intensity of T2-weighted signal on MRI (11–13). Recent studies suggest that female sex, but not age, may impact surgical outcomes. A large retrospective single-center study of 463 patients who underwent TSS found that women were older at surgery, and they had lower pre-operative IGF-I compared to men, although they had larger adenomas and more cavernous sinus invasion than men. Accordingly, rates of total tumor resection were significantly higher in men than in women (92.6% vs. 85.5%; p = 0.021) (11). Another single-center retrospective study of 112 patients similarly showed that women had larger tumors despite lower mean IGF-I levels. However, the postoperative remission rates were comparable in men (51%) and women (56%) and inversely associated with cavernous sinus invasion and GH levels (39). In our study we have also confirmed that clinical characteristics i.e. older diagnosis age (49 vs. 39 years), lower GH, IGF-1 and PRL concentrations at diagnosis, microadenoma on MRI scan as well as lack of imaging features of invasiveness such as extrasellar expansion, optic chiasm compression and invasion of cavernous sinuses increased the chance of surgical remission. The median tumor size in the group of patients who did not obtain surgical cure was almost twice as big as the tumor size in patients with complete removal (23 vs. 12.5 mm). Contrary to the mentioned studies we did not find any significant difference between gender regarding the surgical remission rate. Biermasz et al. (40) showed that early postoperative remission was mainly determined by preoperative GH concentration, not by tumor size. Some other authors defined preoperative GH concentration as the best predictor of remission after TSS using ROC or empiric analysis in a range of 4.5 - 45ng/mL (7 9, 10, 41, 42). We have estimated the preoperative GH concentration cut-off value predicting surgical remission as 8.63 µg/L in our cohort, which is close to the low end of the aforementioned range. Preoperative PRL concentration is one of the discordant factors. Some researchers confirm, that higher PRL concentration is a negative predictor of remission after TSS (43, 44), and other results contradict that (45). In our series we have shown by uni- and multivariate regression analysis that hyperprolactinemia was a negative predictor of surgical remission. Moreover, we have shown that pathomorphological evaluation provides clinicians with another positive predictors of remission after TSS i.e. positive staining for α-SU and DG image of the somatotroph tumor in EM.

Regarding clinical and imaging predictors of medical treatment outcome in acromegaly, the most recent data shows, that older age, higher IGF-1 concentration at diagnosis and hypointense T2-weighted MRI signal increase the chance of better response to first-generation SRLs (19). Störmann et al. (17) indicated also female sex and treatment-näive status as predictors of good response to lanreotide autogel. A meta-analysis of a group of 622 patients showed that lower baseline IGF-1, lower body weight and older diagnosis age were the best predictors of biochemical response to first-generation SRLs (18). In our cohort male sex, lower GH concentration at diagnosis, lack of tumor’s extrasellar expansion, compression of the optic chiasm and cavernous sinuses invasion significantly increased the chance of better response to first-generation SRLs. Lower Ki-67 index tended to increase the chance of good response to SRLs although without reaching statistical significance. We showed also that the features of DG somatotroph tumor in EM were associated with better control during treatment with first generation SRLs. Due to incomplete availability of data, we have not analyzed T2-intensity on MRI scans and SST receptors expression. However, T2-weighted hypointense signal on MRI is proved to be seen nearly exclusively in patients with DG tumors (46, 47), and we did assess the granulation pattern of tumors in EM.

In general, the pathological assessment of tumors’ tissue has increased the value of our research, though we have not analyzed somatostatin receptor expression in postoperative tumor tissues, which might have been a valuable addition to the present study. However, majority of the studied group received preoperative SRLs treatment and it was proved that SSTR2a expression was reduced after octreotide treatment due to down-regulation mechanism so the results should have been interpreted with caution (48).

Over the last years, researchers have been seeking for clinical and molecular predictors of pasireotide response. Coopmans et al. (24) showed that biochemical response to pasireotide-LAR is associated with high T2-signal intensity, whereas patients with a lower SST2 receptor expression have a greater chance to achieve tumor shrinkage. In general, good candidates for pasireotide treatment might be patients poorly responding to first-generation SRLs with high expression of SST5 receptor, with low AIP expression, those with SG tumors and with high Ki-67 index (49). The importance of clinical factors in terms of response to pasireotide-LAR has not been so widely discussed as molecular factors. In the present study we have assessed the influence of clinical, imaging, and pathological characteristics on pasireotide-LAR response but have not found significant differences between good and poor-response groups. Our cohort treated with pasireotide-LAR with available pathological results consisted of 20 patients and is one of the largest compared to previous studies. Nonetheless, division of this group into relatively small subgroups has made the statistical analysis problematic and that may explain why no statistically significant differences were found. Further research on a larger cohort is needed in this field.

Regarding pathological findings affecting treatment outcome, many papers have discussed this topic covering IHC evaluation with pituitary hormones staining, somatostatin receptors expression, E-cadherin expression, Ki-67 index and granulation pattern assessed on the basis of IHC staining for low molecular weight cytokeratins (LMWC) e.g. with the Cam 5.2 antibody. The current study focuses on pituitary hormones staining, Ki-67 index and granulation pattern evaluated in EM. Other elements of pathological assessment were not analyzed due to small groups with available data.

Contrary to Sarkar et al. (50) and Rick et al. (51), who have published different results in terms of remission after TSS rate in somatotroph tumors containing PRL, we have found no differences in treatment effectiveness between pure GH(+), mixed GH(+) PRL(+) and plurihormonal tumors. The only IHC parameter that positively affected surgery outcome in our cohort was α-SU staining. Papers from the 1990s and early 2000s showed, that positive α-SU staining is present in 37% to 58% of acromegalic patients (15, 52) which is similar to our data indicating that 47% patients in our cohort had positive staining for α-SU. Alpha-SU staining is known to occur more commonly in GH(+) PRL(+) tumors (52). Unfortunately, the recent research has rarely raised the meaning of α-SU staining for acromegaly patients not to mention its influence on treatment effectiveness. Waśko et al. (53) has indicated greater decrease in GH and IGF-1 levels after octreotide LAR treatment in patients with higher α-SU serum concentration. The current study is the first one published so far which analyzed correlations between α-SU staining in IHC evaluation and acromegaly treatment outcome. We have demonstrated that acromegaly patients with positive staining for α-SU have a greater chance of surgical remission, but it does not affect the response to first-generation SRLs and pasireotide-LAR. Because staining for pituitary hormones is the basic IHC assessment, positive α-SU staining may be used commonly in clinical practice as one of pathomorphological predictors for remission after TSS, when more advanced methods e.g. granulation pattern assessed on the basis of IHC for LMWC or EM are not available. It is also worth emphasizing that positive staining for the α-SU was actually present only in DG tumors (54).

Another factor increasing the likelihood of surgical remission in our series is Ki-67 index. Some previous data demonstrated that Ki-67 index affects surgical outcome in acromegaly (55, 56) what we have confirmed in a larger group. On the other hand, some authors have not found any association between Ki-67 index and remission after TSS (50, 57–59). Higher Ki-67 index has been associated with tumor’s invasiveness (55, 60, 61) what may explain the lower rate of remission after TSS in patients with higher values of Ki-67 index. The impact of Ki-67 index on medical treatment has also been studied. Kasuki et al. (21) showed, that Ki-67 index is higher in patients without biochemical control of acromegaly on octreotide LAR treatment. This has been confirmed for first-generation SRLs by Fusco et al. (55). However, some papers contradicted these findings, demonstrating no differences in Ki-67 index between hormonally controlled and not-controlled groups on medical treatment, which stands for both first-generation SRLs and for pasireotide-LAR (57, 62). In the current study we have also found no statistically significant difference in Ki-67 indexes in good and poor control groups among patients treated with first-generation SRLs and pasireotide-LAR, although we observed some tendency to higher Ki-67 index in poor-response to first-generation SRLs group. It is worth to mention, that our results concerning the response to medical treatment cannot be easily compared to findings cited above, as we accepted more liberal criteria of good biochemical control on medical treatment.

We have also analyzed association between tumor type in EM and treatment outcome, showing that patients with SG tumors achieve surgical remission less frequently than patients with DG tumors. Despite our data supports some previous papers mentioning surgical outcome (63–65) some other series did not find this association (50, 58, 59). It is worth adding, that our cohort is one of the few assessing granulation pattern and medical treatment outcome. We noted that patients with SG tumors responded worse to first-generation SRLs than patients with DG tumors and comparably well to pasireotide-LAR. Kasuki et al. (21) showed in a group of 24 patients, that DG tumors responded better to first-generation SRLs. Similar results were published by Kiseljak-Vassiliades et al. (64) in a group of 30 patients. On the other hand, Dehghani et al. (65) found no differences in response to first-generation SRLs in a group of 20 patients depending on granulation pattern and so did Iacovazzo (62) in a 39-patient cohort. From Iacovazzo’s et al. group of 39 patients, 11 were treated with pasireotide-LAR and those with SG tumors responded better to pasireotide-LAR. Comparing to the cohorts mentioned above, we have evaluated the association between granulation pattern and medical treatment outcome in a larger group of 62 patients treated with first-generation SRLs and in 20 patients treated with pasireotide-LAR. All authors cited above performed IHC evaluation using Cam5.2 keratin staining for analysis of granulation pattern, which is a substitute for EM used in our study. The WHO classification from 2017 considers SG somatotroph tumors as high-risk tumors with potential aggressive behavior (54, 66). Our results might suggest greater resistance to treatment of SG somatotroph tumors in terms of lower chance of surgical remission and poorer response to first-generation SRLs in patients with such tumors. However, the aggressiveness of SG somatotroph tumors defined as uncontrolled growth remains disputable. Probably that was the reason, why the WHO classification from 2022 does not include SG somatotroph tumors into high-risk tumors (67).

Our study has some limitations, as its retrospective design contributed to incomplete data in some patients. Furthermore, we included patients treated surgically within 20 years, which resulted in different patterns of pathological evaluation over the timespan (e.g. SST receptors expression was not available in majority of patients and thus not included in the final analysis). A similar situation occurs in the case of GH and IGF-1 assays, which changed over the time. What is more, the tissues were evaluated by one pathologist for the purposes of this study, which may introduce the observer bias. On the other hand, the strength of our study is a relatively large study cohort managed in one clinical center. As shown above, we have presented one of the largest group of patients with pathological assessment of somatotroph tumor sample and its impact on pharmacological treatment published so far.

## Conclusions

The current study shows that GH, IGF-1 and PRL concentration, tumor size and invasiveness, as well as granulation pattern may predict surgical outcome in acromegaly, whereas male sex, GH concentration and granulation pattern may also forecast the response to first-generation SRLs. Staining for α-SU, routinely assessed in IHC evaluation, was shown to be positive more frequently in patients, who achieved surgical remission. Our results confirm the importance of combination of hormonal, imaging and pathological characteristics in acromegaly patients’ evaluation. These factors provide clinicians with valuable information on expected treatment outcome, therefore play a significant role in personalized treatment planning in patients with acromegaly. Further study searching for the best models predicting acromegaly remission, which would combine clinical and modern molecular parameters is needed in larger patients’ cohorts.

## Data availability statement

The datasets presented in this article are not readily available because the dataset comes from the Polish Acromegaly Registry. The privacy policy and personal data protection rules assume only results sharing and not dataset sharing. Requests to access the datasets should be directed to Agnieszka Tomasik, agnieszka@tomasik.pl.

## Ethics statement

The studies involving human participants were reviewed and approved by the Bioethics Committee of the Centre of Postgraduate Medical Education in Warsaw, Poland. The patients/participants provided their written informed consent to participate in this study.

## Author contributions

AT, MS-B, IC-O, GZ, JK and WZ were involved in managing the patients. MM did the pathological evaluation. AT, MS-B, MM, IC-O were involved in collecting and analysing the data. DR did the statistical analysis. AT, MS-B, MM, IC-O and DR wrote and revised the manuscript. GZ, JK and WZ revised the manuscript. All authors approved the final version of the manuscript.

## Funding

This study is a part of project, which received funding from IPSEN, Pfizer and Recordati. The funders were not involved in the study design, collection, analysis, interpretation of data, the writing of this article or the decision to submit it for publication.

## Conflict of interest

The authors declare that the research was conducted in the absence of any commercial or financial relationships that could be construed as a potential conflict of interest.

## Publisher’s note

All claims expressed in this article are solely those of the authors and do not necessarily represent those of their affiliated organizations, or those of the publisher, the editors and the reviewers. Any product that may be evaluated in this article, or claim that may be made by its manufacturer, is not guaranteed or endorsed by the publisher.
